# The Spectrum of Genetic Defects in Chronic Lymphocytic Leukemia

**DOI:** 10.4084/MJHID.2012.076

**Published:** 2012-11-13

**Authors:** Davide Rossi, Marco Fangazio, Gianluca Gaidano

**Affiliations:** 1 Division of Hematology, Department of Translational Medicine, Amedeo Avogadro University of Eastern Piedmont, Novara, Italy

## Abstract

Chronic lymphocytic leukemia (CLL) is the most common leukemia in the Western world and shows a remarkable heterogeneity in the clinical course. Understand the genetic basis of CLL may help in clarifying the molecular bases of this clinical heterogeneity. Recurrent chromosomal aberrations at 13q14, 12q, 11q22–q23 and 17p13, and *TP53* mutations are the first genetic lesions identified as drivers of the disease. While some of these lesions are associated with poor outcome (17p13 deletion, *TP53* mutations and, to a lesser extent, 11q22–q23 deletion) others are linked to a favorable course (13q14 deletion as sole aberration). Recently, next generation sequencing has revealed additional recurrent alterations in CLL targeting the *NOTCH1*, *SF3B1*, and *BIRC3* genes. *NOTCH1*, *SF3B1*, and *BIRC3* lesions provide: *I)* new insights on the mechanisms of leukemogenesis, tumor progression and chemoresistance in this leukemia; *II)* new biomarkers for the identification of poor risk patients, having individually shown correlations with survival in CLL; and *III)* new therapeutic targets, especially in the setting of high risk disease. This review will summarize the most important genetic aberrations in CLL and how our improved knowledge of the genome of leukemic cells may translate into improved patients' management.

## Introduction

In Western countries, chronic lymphocytic leukemia (CLL) is the most frequent mature B-cell malignancy.^1^,^2^ The course CLL ranges from very indolent, with a nearly normal life expectancy, to rapidly progressive leading to early death.^3^–^8^ Understand the genetic basis of CLL may help in clarifying the molecular determinants of this clinicalclinical heterogeneity and improve patients' prognostication.

Recurrent chromosomal aberrations at 13q14, 12q, 11q22–q23 and 17p13 are the first genetic lesions identified as drivers of the disease, and has enabled the construction of a hierarchical model of cytogenetic abnormalities that correlates with outcome.^9^ Cytogenetic lesions, however, may not entirely explain the genetic basis of CLL clinical heterogeneity, as documented by the contribution of *TP53* mutation assessment in identifying high risk patients.^9^ The recent major improvements in massive parallel sequencing technologies have provided an opportunity to examine the CLL genome, allowing for the identification of genomic alterations underlying the disease and for the discovery of new therapeutic targets and clinically predictive biomarkers such as *NOTCH1*, *SF3B1* and *BIRC3*.^10^–^16^

## Prevalence of Genetic Lesions at Different CLL Clinical Phases

During its history, CLL may proceed through distinct clinical phases, ranging from a pre-malignant condition known as monoclonal B-cell lymphocytosis (MBL), to overt CLL, and even transformation into an aggressive lymphoma (Richter syndrome).^1^,^2^

Similarly to other pre-malignant conditions, also MBL frequently harbor genetic changes that can be found in the overt disease. In MBL, 13q14 deletion occurs at the same prevalence as in overt CLL (~40–50% of cases), even when the number of circulating monoclonal CLL-like cells is extremely small, thus indicating that this lesion occurs early during the natural history of the disease.^17^–^21^ What distinguishes MBL from CLL is the rate of occurrence of genetic lesions that are considered secondary events and that associate with poor outcome in this leukemia.^19^,^21^ In clinical MBL, 11q22–q23 deletion, 17p13 deletion and mutations of *BIRC3*, *TP53*, *NOTCH1* and *SF3B1* may be observed in ~1–3% of cases, a prevalence that is significantly lower than that of CLL (Table I).^17^,^19^,^21^,^22^ High risk cytogenetic abnormalities have been occasionally described also in low count MBL, but the biological implications of this observation are currently unknown.^18^,^20^

When CLL is overt, three major clinical phases can be envisaged, including: *I)* newly diagnosed CLL; *II)* progressive CLL; and *III)* relapsed and fludarabine-refractory CLL (Table I).^2^*TP53* abnormalities, including mutations and 17p13 deletions, are observed in ~5–10% newly diagnosed CLL, in ~10% progressive CLL requiring first treatment,^9^,^23^–^32^ and in ~40–50% relapsed and fludarabine-refractory CLL,^33^–^35^ thus representing the most frequent lesions in this high risk clinical condition. Deletion of 11q22–q23 occurs in 10–15% in newly diagnosed CLL, 9,36 while its prevalence raises to 20–25% at the time of first treatment and 25–30%% at fludarabine-refractoriness.^24^,^29^,^33^,^34^ Mutations of *ATM*, which is included in the minimal common region of deletion on 11q22–q23, have been shown to be present in 12% of newly diagnosed patients and in 15% progressive CLL requiring first treatment.^37^–^40^ By combining mutations and deletions, genetic lesions of *ATM* occur in 25% of diagnostic samples of CLL and in 37% cases requiring first treatment.^37^–^40^ These frequencies make *ATM* alterations the most common genetic lesions predicting poor outcome at CLL presentation and treatment requirement.

Among the novel genetic alterations disclosed by whole genome/exome sequencing, *NOTCH1*, *SF3B1* and *BIRC3* lesions follows the same distribution across CLL clinical phases as *TP53* and *ATM* abnormalities (Table 1). *NOTCH1* mutations recur in ~10% unselected newly diagnosed CLL while their prevalence increases to 15–20% in progressive and relapsed cases.^10^,^11^,^14^*SF3B1* mutations have been identified in ~7% unselected newly diagnosed CLL, while their prevalence rises to 17% in relapsed and fludarabine-refractory patients.^12^,^13^,^16^*BIRC3* lesions occur at low rate (4% of cases) in unselected newly diagnosed CLL, while are enriched among relapsed and fludarabine-refractory CLL (24% of cases).^15^ Because of their recent identification and the lack of information from large clinical trials, the precise rate of occurrence of *NOTCH1*, *BIRC3*, and *SF3B1* lesions at the time of first treatment requirement still remains to be clarified.

Within the spectrum of the various aspects of CLL, Richter syndrome (RS) is the most aggressive clinical phenotype because of the combined effect of chemoresistance and rapid disease kinetics. The clinical behavior of RS is strongly related to its genetic background (Table I). The high rate of *TP53* abnormalities, which occur in ~60% cases and represent the most frequent genetic lesion at the time of transformation, accounts for the chemoresistance that is very common in RS.^41^*NOTCH1* mutations are the second most frequent genetic lesion in RS, where they occur in ~30% of cases.^10^ Among the other high risk genetic lesions, *ATM* abnormalities, *BIRC3* genetic lesions and *SF3B1* mutations that are otherwise enriched at the time of chemorefractoriness are rare or absent in RS, thus strengthening the notion that RS is molecularly distinct from chemorefractory progression without transformation.^13^,^14^,^41^

### *TP53* Abnormalities

The tumor suppressor gene *TP53* codes for a central regulator of the DNA-damage-response pathway, and its activation leads to cell-cycle arrest, DNA repair, apoptosis, or senescence through both transcription-dependent and transcriptional-independent activities.^42^ Among CLL harboring *TP53* abnormalities, mutations of *TP53* co-occurred with deletion of the corresponding locus in ~70% of cases, consistent with a dual hit mechanism of inactivation.^43^ The remaining ~30% of cases have 17p13 deletion in the absence of *TP53* mutations (~20%), or *TP53* mutations in the absence of 17p13 deletion (~10%). *TP53* mutations are mainly represented by missense substitutions targeting the DNA-binding domain, while the remaining are truncating lesions. Mutations either directly disrupt the DNA binding domain of *TP53* or cause conformational changes of the TP53 protein, thus leading to severely impaired TP53 function.^43^,^44^

The clinical importance of *TP53* abnormalities in CLL is tightly linked to their close association with poor outcome and refractoriness, as documented by a number of observational studies and prospective trials led in both the chemotherapy and immuno-chemotherapy era. Among unselected newly diagnosed CLL, patients harboring 17p13 deletion have an estimated median overall survival (OS) of only 3–5 years.^9^,^45^ However, it is important to stress that there is a small subgroup of patients with 17p13 deletion (and mostly mutated immunoglobulin genes) who may exhibit stable disease for years without treatment indications.^45^

The outcome of patients with 17p13 deletion and need for treatment is very poor. With the most effective regimen available today for CLL, i.e. FCR (fludarabine-cyclophosphamide-rituximab), patients with 17p13 deletion have a poor response (5% of complete response vs ~50% in non 17p13 deleted CLL), a short progression free survival (PFS) (11.2 months vs 51.8 months) and OS (38.1% at 36 months).^29^ This is in line with the established importance of the wild-type TP53 protein in mediating the cytotoxicity of DNA-damaging agents including purine analogs.

A number of prospective studies suggest that, in addition to 17p13 deletion, also *TP53* mutations, even in the absence of 17p13 deletion, predict poor outcome in CLL. In the GCLLSG CLL4 trial (fludarabine vs fludarabine-cyclophosphamide) no complete response were observed in *TP53* mutated CLL, and the median PFS (23.3 vs 62.2 months) and OS (29.2 vs 84.6 months) were significantly shorter in the group with *TP53* mutation.^30^ In the GCLLSG CLL8 trial (fludarabine-cyclophosphamide vs FCR), patients with *TP53* mutations showed the lowest complete response and overall response rates (6.9% vs. 36.4% and 62.1% vs. 95.3%), translating into shorter PFS (12.4 months vs. 45 months) and OS (39.3 months vs not reached in all other patients).^44^ In the UK LRF CLL4 trial (chlorambucil vs fludarabine vs fludarabine-cyclophosphamide), the complete response rate of *TP53* mutated patients was only 5% with a 5-years PFS of 5% and a 5-years OS of 20%.^31^

Based on these data, 17p13 deletion is the sole cytogenetic abnormality that is recommended to be tested by FISH in CLL patients requiring treatment.^2^ Since CLL with *TP53* mutations experience poor prognosis regardless of the presence of 17p13 deletion, the *TP53* mutation analysis should be integrated into the evaluation of CLL patients before treatment initiation.^44^ CLL patients carrying *TP53* alterations, regardless of whether mutated or deleted, should be redirected to different therapeutic regimens compared to the standard chemo/chemoimmuno-therapies.^2^,^33^,^35^,^44^,^46^

### *NOTCH1* Mutations

The *NOTCH1* gene encodes a heterodimeric transmembrane protein that functions as a ligand-activated transcription factor with a high conserved pathway.^47^ When the NOTCH1 receptor interacts with its ligands through the extracellular subunit, two consecutive proteolytic cleavages of the protein are initiated and lead to pathway activation.^47^,^48^ The S2 cleavage in the heterodimerization domain is performed by ADAM10, and is followed by the S3 cleavage by the γ-secretase complex. Upon activation the cleaved intracellular portion of NOTCH1 (ICN) translocates into the nucleus where it modifies the expression of target genes, including the *MYC* oncogene. As a transcriptional factor, *NOTCH1* plays an important role in a number of cellular functions during embryogenesis and in self-renewing tissues of the adult organism, including maintenance of stem cells, cell fate specification, proliferation, and apoptosis.^48^ One of the mechanisms of the NOTCH1 signal suppression is operated through the PEST [proline (P), glutamic acid (E), serine (S), and threonine (T) rich] domain that directs the activated NOTCH1 towards proteosomal degradation.^47^ A major role of *NOTCH1* in lymphoid cells in the adult organism is the commitment of hematopoietic progenitors to differentiate toward T lineage.^49^ Conversely, in mature B-lymphocytes, *NOTCH1* signaling promotes terminal differentiation to antibody-secreting cells.^50^

*NOTCH1* mutations were the first molecular lesion identified through massive parallel next generation sequencing in CLL by two independent groups.^10^,^11^*NOTCH1* mutations are significantly more frequent in CLL with unmutated, rather than mutated, immunoglobulin genes, are significantly enriched in CLL harboring trisomy 12, and identify a distinct clinico-molecular subgroup of CLL with deregulated cell cycle and short survival.^10^–^12^,^14^,^16^,^51^–^53^

**Figure 1:**
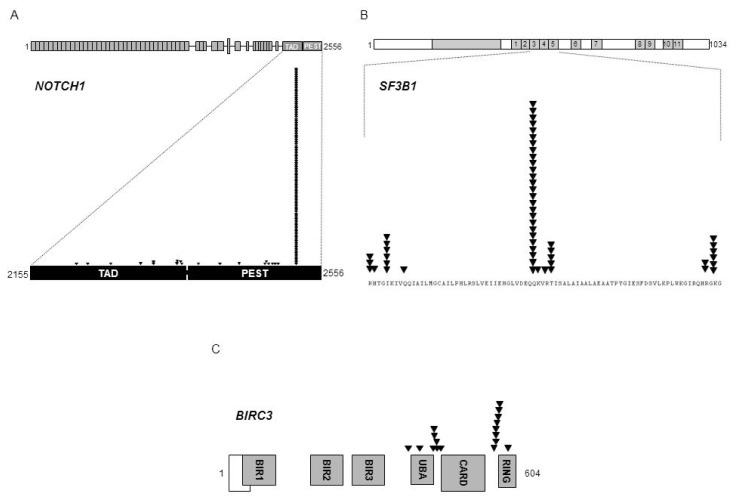
*NOTCH1*, *SF3B1*, and *BIRC3* mutation distribution in CLL. Schematic representation of the human *NOTCH1* (panel A), *SF3B1* (panel B), and *BIRC3* (panel C) proteins, with their key functional domains. Symbols indicate the position of the mutations. Mutations are from the Novara CLL mutation database and from the COSMIC database (v61).

*NOTCH1* mutations in CLL mainly clusters within a hotspot in exon 34, and are commonly represented by a single 2-bp deletion (c.7544_7545delCT) that accounts for ~80–95% of all *NOTCH1* mutations in this leukemia (Figure 1).^10^–^12^,^14^,^16^,^51^–^53^ The predicted functional consequence of *NOTCH1* mutations in CLL is the disruption of the C-terminal PEST domain resulting in activated NOTCH1 protein, impaired degradation and accumulation, and sustaining deregulated signaling.^11^ Consistent with this notion, a number of cellular pathways are specifically altered in CLL harboring *NOTCH1* mutations.^11^,^52^

Beside their pathogenetic role, *NOTCH1* mutations also represent a new biomarker for the identification of poor risk CLL patients. *NOTCH1* mutated patients have a rapidly progressive disease and a significantly shorter survival probability (21–45% at 10 years) compared to *NOTCH1* wild type cases (56–66% at 10 years).^10^,^11^,^14^ The poor prognosis associated with *NOTCH1* mutations in CLL may be explained, at least in part, by a substantial risk (~40–50%) of developing Richter syndrome.^10^,^11^,^14^

*NOTCH1* is a potential therapeutic target in CLL. Treatment with γ-secretase inhibitors induces apoptosis of CLL cells by inhibiting the enzymatic S3 cleavage necessary for NOTCH1 activation.^47^,^54^,^55^ However, the limitations due to toxicity of γ-secretase inhibitors in the clinical setting suggest that alternative strategies may be needed for the therapeutic targeting of NOTCH1.

### *SF3B1* Mutations

The spliceosome machinery, a complex of five small nuclear ribonucleoproteins (snRNPs), contributes to the formation of mature mRNA through the removal of introns in the precursor messenger RNA (pre-mRNA) of protein-encoding genes, and is involved in both normal and alternative splicing.^56^ Alternative splicing can generate numerous transcript variants from a single gene, contributing to genomic complexity and potentially to cancer.^57^

*SF3B1* is a core component of the U2 snRNP that recognizes the 3′ splice site at the intron-exon junctions.^56^,^58^–^61^ Structurally, the SF3B1 protein has two well-defined regions: *I)* the N-terminal amino acid region which contains several protein-binding motifs and functions as a scaffold to facilitate its interaction with other splicing factors; and *II)* the C-terminal region which contains 22 non-identical tandem repeats of the HEAT motif that meander around the SF3b complex.^56^,^58^–^61^

Whole genome/exome sequencing technologies allowed for the identification of *SF3B1* as a recurrently mutated gene in CLL.^12^,^13^,^16^*SF3B1* mutations in CLL cluster in selected HEAT repeats of the SF3B1 protein, target a number of hotspots (codons 662, 666, 700, 742), and are generally represented by missense substitutions (Figure 1).^12^,^13^,^16^ Notably, an identical spectrum of *SF3B1* mutations has been identified in other hematopoietic tumors of the myeloid compartment.^62^

The precise biological consequences of *SF3B1* mutations in CLL are currently unknown. However, the clustering of *SF3B1* mutations within the HEAT domains suggests that they are selected to modify SF3B1 interactions with other proteins of the spliceosome complex, thus resulting in deregulated normal and alternative mRNA splicing.^12^,^16^

Consistent with their accumulation in the more advanced phases of the disease, *SF3B1* mutated patients show a significantly shorter overall survival (34–48% at 10 years) compared to wild type cases (60–73% at 10-years).^12^,^13^,^16^

### *BIRC3* Abnormalities

In CLL, activation of the NF-κB pathway contributes to the acquisition of a chemorefractory clinical phenotype and correlates with poor outcome.^63^–^67^ The Baculoviral IAP repeat containing 3 (*BIRC3*) gene is one of the components of a protein complex that negatively regulates the MAP3K14 serin-threonine kinase, the downstream activator of non-canonical NF-κB signaling.^63^–^66^

*BIRC3* was found to be recurrently disrupted by mutations, deletions, or a combination of mutations and deletions in CLL patients.^15^*BIRC3* inactivating mutations and a fraction of *BIRC3* deletions cause a truncation of the C-terminal RING domain of the BIRC3 protein, essential for ubiquitination, and the following proteasome degradation, of MAP3K14, and drives constitutive non-canonical NF-κB activation (Figure 1).^15^

The *BIRC3* gene maps to 11q22.2, approximately 6Mb centromeric to the *ATM* locus. The identification of *BIRC3* involvement in CLL might be important for elucidating the molecular genetics of 11q22–q23 deletion, a frequent cytogenetic abnormality predictive of poor outcome. In fact, although *ATM* has been regarded as the relevant gene of this chromosomal abnormality, biallelic inactivation of *ATM* does not exceed ~30% of cases with 11q22–q23 deletion.^36^–^39^ The presence of an additional tumor suppressor in the 11q22–q23 region has been postulated,^40^ and *BIRC3* implicates a suitable candidate.

From a clinical standpoint, *BIRC3* lesions contribute to clinical aggressiveness and fludarabine refractoriness in CLL.^15^ Indeed, *BIRC3* lesions identify a subgroup of CLL displaying poor survival (median 3.1 years) similar to that associated with *TP53* abnormalities.^15^

In CLL, fludarabine refractoriness may be explained by *TP53* disruption in ~40% of patients, while ~60% high risk CLL do not present *TP53* abnormalities.^34^ Intriguingly the distribution of *BIRC3* disruption and *TP53* abnormalities is mutually exclusive and *BIRC3* abnormalities can recapitulate the genetics of ~40% chemorefractory and *TP53* wild type CLL.

On these bases, *BIRC3* disruption may contribute to expand the panel of biomarkers for the early identification of chemorefractory cases.^15^ In addition, *BIRC3* abnormalities provide a molecular rationale for targeting NF-κB in poor risk and chemorefractory CLL. NF-κB inhibitors are under development in CLL and pre-clinical findings suggest that these compounds might be active against chemoresistant CLL clones.^67^,^68^

## Figures and Tables

**Table 1:** Prevalence of CLL recurrent lesion stratified according the disease phase

	*TP53* disruption	del 11q22–q23	*NOTCH1* mutations	*SF3B1* mutations	*BIRC3* disruption
MBL	1–2%	0–3%	3%	1–2%	0
Diagnosis	5–10%	10–15%	8–11%	4–7%	0.05
First treatment	10–11%	20–25%	10–15%	17%	n.a.
Chemorefractoriness	40–50%	25–30%	15–20%	17%	25%
Richter Syndrome	50–60%	10%	30–40%	6%	0

CLL, Chronic lymphocytic leukemia; MBL, Monoclonal B-cell lymphocytosis
